# Therapeutic Adherence of People with Mental Disorders: An Evolutionary Concept Analysis

**DOI:** 10.3390/ijerph20053869

**Published:** 2023-02-22

**Authors:** Carlos Laranjeira, Daniel Carvalho, Olga Valentim, Lídia Moutinho, Tânia Morgado, Catarina Tomás, João Gomes, Ana Querido

**Affiliations:** 1 School of Health Sciences, Polytechnic of Leiria, Campus 2, Morro do Lena, Alto do Vieiro, Apartado 4137, 2411-901 Leiria, Portugal; 2 Centre for Innovative Care and Health Technology (ciTechCare), Polytechnic of Leiria, Rua de Santo André—66–68, Campus 5, 2410-541 Leiria, Portugal; 3 Comprehensive Health Research Centre (CHRC), University of Évora, 7000-801 Évora, Portugal; 4 Hospital Center of Leiria–Hospital de Santo André, R. de Santo André, 2410-197 Leiria, Portugal; 5 Group Innovation & Development in Nursing (NursID), Center for Health Technology and Services Research (CINTESIS@RISE), 4200-450 Porto, Portugal; 6 Nursing School of Lisbon (ESEL), Av. Prof. Egas Moniz, 1600-096 Lisboa, Portugal; 7 Nursing Research, Innovation and Development Centre of Lisbon (CIDNUR), Av. Prof. Egas Moniz, 1600-096 Lisboa, Portugal; 8 Pediatric Hospital, Coimbra Hospital and University Centre, R. Dr. Afonso Romão, 3000-602 Coimbra, Portugal; 9 The Health Sciences Research Unit: Nursing (UICISA: E), Nursing School of Coimbra (ESEnfC), 3004-011 Coimbra, Portugal

**Keywords:** concept analysis, mental health, therapeutic adherence, ecological approach

## Abstract

Patient therapeutic adherence lies at the core of mental health care. Health Care professionals and organizations play a major role in promoting adherence among people with mental disorders. However, defining therapeutic adherence remains complex. We used Rodgers’ evolutionary concept analysis to explore the concept of therapeutic adherence in the context of mental health. We conducted a systematic literature search on Medline/PubMed and CINAHL for works published between January 2012 and December 2022. The concept analysis showed that major attributes of therapeutic adherence include patient, microsystem and meso/exosystem-level factors. Antecedents are those related to patients, such as their background, beliefs and attitudes, and acceptance of mental illness–and those related to patient-HCP therapeutic engagement. Lastly, three different consequences of the concept emerged: an improvement in clinical and social outcomes, commitment to treatment, and the quality of healthcare delivery. We discuss an operational definition that emerged from the concept analysis approach. However, considering the concept has undergone evolutionary changes, further research related to patient adherence experiences in an ecological stance is needed.

## 1. Introduction

In 2019, mental health disorders impacted around 970 million individuals globally, mostly with anxiety and depressive conditions [1], corresponding to 14% of the global illness burden and 30% of the non-fatal disease burden. These numbers are exacerbated by therapeutic nonadherence [2]. A recent meta-analysis found that rates of medication nonadherence in bipolar disorder, major depression, and schizophrenia were 44%, 50%, and 56%, respectively [2]. Assessing therapeutic adherence in clinical care is essential because nonadherence results in poor outcomes in mental health disorders worldwide [3]. 

In its 1995 report, the National Council on Patient Information and Education (NCPIE) described adherence as ‘following a medicine treatment plan developed and agreed on by the patient and his/her health professional(s)’ [4] (p. 10). To avoid the paternalistic connotation of the term “compliance”, the World Health Organization (WHO) introduced the term “adherence”, used mostly in the fields of psychology and social sciences [5]. This term emphasizes that acceptance of professional recommendations relies on the patient’s own decisions. According to Christensen et al. [6], social scientists tend to use the term “adherence”, whereas pharmacists use the term “compliance”. Although both terms are commonly used interchangeably [7], Bissonnette’s [8] definition of adherence includes the concept of compliance, stressing the need for patient consent and attributing greater accountability to the therapist in the development of a trusting relationship between Health Care Professional (HCP) and patient. Indeed, the term “adherence” respects patient views while also recognizing that merely receiving medication is not a very effective strategy [7,9]. Furthermore, adherence requires patients to have an active part in developing a therapeutic connection with their HCP. Recent evidence supports that adherence to medication depends upon personality traits and beliefs and perceptions of mental illness [10,11]. A systematic review has found that adherence to psychopharmacological treatment is associated with patient characteristics (sociodemographic and clinical variables), health beliefs and psychological variables, such as self-efficacy and locus of control [12]. 

In this context, therapeutic adherence is now consensually accepted as fundamental for the optimal management of any disease and interrelated to high levels of health literacy [13]. Despite the recognized importance of adherence and the scientific community’s great attention in recent decades, nonadherence remains a serious public health problem, linked more to the economic costs of the disease than just the associated human costs. Additionally, treatment complexity (polypharmacy) and illness chronicity have been identified as variables negatively associated with nonadherence to psychopharmacological treatment [12]. Gardner [14] presents adherence as a complex multidimensional concept encompassing a wide range of factors such as health system, social/economic factors and condition-related, therapy-related, and patient-related factors. To express the pivotal place of patients and carers in shared decision making and adherence to the therapeutic regimen, a new perspective of therapeutic management has arisen from a person-centered approach focused on empowerment.

Despite decades of using the terms “adherence”, “compliance”, and “self-management” in the literature, there is still a lack of conceptual clarity. While comparable in meaning, each term has been defined differently across fields. This paper provides a conceptually adequate definition of therapeutic adherence to encourage optimal experiences and outcomes for mental health patients. This approach offers a systematic and dynamic method to assess evolutionary changes and transdisciplinary variations in the meaning of concepts [15].

### 1.1. Background

Several studies have shown that patients increasingly want to feel included in the clinical process, want to obtain more information and demand greater interaction with HCPs [16]. This calls for a bio-psychosocial approach that sees patients as active partners in therapy. In this sense, the HCP diagnoses the person’s health situation and evaluates the family dynamics, taking the opportunity to promote healthy lifestyles and health potential through the optimization of the processes of life, growth and development, and to provide information that generates learning and enables people to reach their maximum health potential. Nonadherence should be understood as individual responses to the lack of coincidence between their ideas and those of the HCP regarding their problems and/or treatments.

The lack of adherence occurs, therefore, when the patient’s behavior does not coincide with the HCP’s recommendations. The concept’s definition is not limited to deviations in the application of the prescribed therapeutic regimen, but also includes failure to follow directions relating to changes in health habits and lifestyles and adoption of healthy practices, as well as not performing additional diagnostic tests and missed mental care appointments. The most common reasons reported include “the interval between scheduling and appointment day, forgetting, being discharged against medical advice, and problems with substance abuse” [17] (p. 177).

Given the important repercussions of a lack of adherence on public health, research has attempted to measure the dimension of the phenomenon. Medical prescriptions are intended to bring benefits to the patient, however, their incorrect use by the patient can have consequences for themselves and wider social and economic effects. Indeed, the lack of adherence to therapy can result in poor clinical outcomes and worsening patient health status [18], eventually causing errors in diagnosis and treatment. This deterioration in clinical condition may also require the subsequent prescription of more drugs, new diagnostic or therapeutic procedures that are more costly and complex, more consultations, the use of emergency services, higher (re)hospitalization rates, increased health care expenses, and increased suicide [1]. Conversely, improved adherence over time relates to improved quality of life in severe mental illnesses [19].

Therapeutic adherence is affected by the context in which a patient is expected to adhere to the prescribed treatment, such as connections with family/friends and community, and additional social aspects. These factors frequently occur concurrently and interact reciprocally. The Person-Centered Care and Communication Continuum (PC4 Model) was recently designed to educate HCPs regarding care practices, discourse settings, and communication contents and forms that might increase or hinder patient-centered care in clinical practice [20]. These professionals must be aware of how their communication—whether directed toward job completion, following care protocols, or addressing the needs of patients and caregivers—can affect patient-centered care. HCPs must be aware of the care context, the patients’ particular conditions, as well as their nonverbal language and actions.

Subjective norms, perceived behavioral control, attitudes and behavioral intention, are four Planned Behavior Theory qualities that form a framework for predicting why patients modify their behavior [21]. Fernandez-Lazaro et al. [22] emphasized that therapeutic adherence demands voluntary actions and participation since it is a commitment to manage the disease by adhering to the HCP’s treatment recommendations. Furthermore, because patient behavior depends on the system in which patients live and get health care, theories that incorporate system-level factors may more thoroughly explain therapeutic adherence behavior. The system’s value in fostering behavioral changes cannot be overstated [23]. Ecological models, such as Bronfenbrenner’s seminal work on human development, provide a framework for explaining the effect of healthcare systems on patient behavior (e.g., adherence) [24]. In this ecological approach, several layers of variables impact patient behavior, including influences at the patient, microsystem (provider and social support), meso/exosystem (health care organization), and macrosystem levels (cultural and social values). Nonetheless, the last three levels have not been studied as thoroughly as patient-level issues. To fully tackle therapeutic adherence, these elements must be considered in conceptualization and intervention regarding adherence.

Accordingly, there is a need to expand the understanding of therapeutic adherence in mental healthcare. The examination of the antecedents, attributes, and consequences related to the concept will provide greater awareness of barriers and facilitators that affect the desired adherence behavior.

### 1.2. Aim and Research Questions

This review aimed to describe the findings of an evolutionary method concept analysis [25] of the term “therapeutic adherence” in the mental health context, particularly for adults with mental disorders. Thus, the following two questions were addressed:

1. What surrogate terms and related concepts are used?
2. What attributes, antecedents, and consequences apply to the concept of therapeutic adherence in the mental health context?

## 2. Materials and Methods

### 2.1. Study Design

The six steps of Rodgers’ evolutionary method [25] of concept analysis were used to analyze the data and identify attributes, surrogate and related terms, antecedents, and consequences of the therapeutic adherence concept.

### 2.2. Literature Search Methods

A systematic search was conducted using combined search terms ((Mental Disorders OR Mental Health OR Mental illness OR psychiatr*)) AND ((Therapeutic Adherence OR Treatment Adherence OR Adherenc* OR Treatment Adherence and Compliance OR Therapeutic Compliance OR Medication Compliance OR Management, Disease OR Medication Therapy Management OR Patient Compliance OR Compliance with Medical Regimen)) AND (((Psychiatric Rehabilitation OR recovery OR Services, Mental Health)) AND (Shared decision making)) within the titles or abstracts in online databases Medline/PubMed and CINAHL. To establish the contemporary concept operation and meaning, the results were limited to the English and Portuguese languages and restricted to the years 2012–2022.

Studies were included in this review (a) if their focus was therapeutic adherence in the mental health context; and (b) they were related to adults with mental health disorders and/or HCPs who care for these patients. The study excluded articles if they (a) were not particularly connected to mental health care and (b) did not involve primary research. The search was additionally supplemented with reference searches in included literature. The Preferred Reporting Items for Systematic Reviews and Meta-Analyses (PRISMA) statement was followed in this review [26].

**Figure 1:**
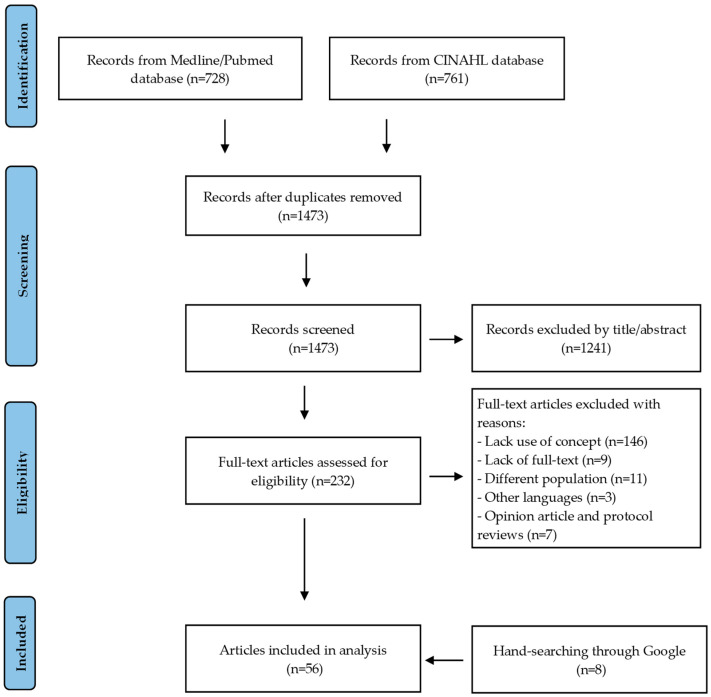
Data search and selection process using the PRISMA flow chart.

Figure 1 depicts the results of the searches and the articles chosen for this analysis. After accounting for duplicate publications, 1473 of the 1489 papers obtained were reviewed, and 232 were appraised using full text. Of these 232 articles, 184 were excluded because they did not meet the inclusion criteria. Additionally, hand-searching through Google was used to identify relevant articles that had not been tagged in our initial search. Following the screening procedure, 56 articles were deemed relevant and included in the concept analysis. These articles addressed a range of disciplines, including nursing, psychology, medicine, pharmacy, and public health, as well as a wide range of healthcare settings, including inpatient, outpatient, and community care. The diversity of this literature reinforces the applicability of our conclusions across a wide range of scenarios. The reference management software Mendeley was utilized to examine each study using a title-first approach and to get the relevant records for abstract screening.

## 3. Results

The results were classified and sequenced using the evolutionary technique [15]. Surrogate terms use alternate words or terminology to express the concept under consideration, and related terms are conceptually distinct but share certain properties with the concept. Antecedents are preceding elements and conditions that are required for therapeutic adherence, although these elements are insufficient on their own. Consequences are the outcomes of the combined operation of attributes [25].

### 3.1. Concept of Interest, Surrogate Terms, and Related Concepts

The World Health Organization [5] tried to standardize the concept of “therapeutic adherence” as the extent to which a person’s behavior–taking medication, following a diet, and/or executing lifestyle changes–corresponds to recommendations agreed with a healthcare provider. Balkrishnan [27] proposed a similar definition, based on the extent to which a patient participates in an agreed-upon treatment regimen. Both definitions stress the need for patients to be engaged and committed to treatment decisions. The concept of adherence presupposes an active, voluntary, and collaborative behavior by the patient, who should be fully aware of their health and is committed to what they should do.

Grady and Gough [28] suggest that self-management is a surrogate term, described as a basic set of skills for handling activities that an individual must perform on a regular basis to regulate or decrease the impact of a medical condition on their lives. Self-management frequently includes a combination of generic and condition-specific components, such as managing the treatment regimen, emotions, and (new) life responsibilities. It goes beyond education and includes assisting patients in actively recognizing and solving problems connected with their illness [28].

The literature search also revealed four terms related to “therapeutic adherence”: compliance, concordance, cooperation, maintenance, commitment, and engagement.

Compliance, the oldest term (the 1970s of the last century), is defined as “the extent to which the patient’s behavior matches the prescriber’s recommendations” [7] (p. 32). This concept of compliance appears closely linked to strict compliance with medical prescriptions and a doctor-patient relationship based on the patient passively and submissively following the doctor’s orders. This paternalistic and unequal perspective of the patient-HCP relation, in addition to blaming the patient, does not describe all the aspects that can affect compliance, particularly in chronic diseases.

In the late 1990s, patients became more active in managing their illnesses and participating in health care in general. The cultural shift from the role of the submissive patient to a more egalitarian view of the HCP-patient/user relationship promoted the decline of the term “compliance”, even leading the Royal Pharmaceutical Society of Great Britain to recommend replacing the term “compliance” with “concordance” [7,29]. Although this implied a new approach to the HCP-patient relationship regarding an agreement on the therapeutic regimen, the change in terminology was not accepted by many researchers and health professionals, leading to alternative terms, such as “cooperation” [30]. The latter quickly gained popularity because it seems to embody the notions of compliance and concordance. In addition, some authors suggested another dimension, “maintenance”, which refers to the self-care tasks that occur when the patient incorporates the treatment into their lifestyle [31]. Moreover, the concepts of “commitment” and “engagement” to treatment were recently developed and used [32]. Both terms are aligned with a person-centered approach focused on empowerment.

### 3.2. Attributes

The main attributes synthesized in this analysis represent the core features of therapeutic adherence, at the individual (patient characteristics), microsystem (family, providers and social attributes), and meso/exosystem levels (health care organization and other contexts having an indirect influence on the person/interrelation of systems) (see Table 1).

In all analyzed studies, the process of adherence is dynamic rather than static, involving objective and subjective personal experiences characterized by the ability to establish interpersonal skills [33]. At the individual level, this process includes the patient’s readiness to change and motivation for mental health treatment [34,35,36,37], their medication literacy (readiness to understand the role of medication, how to use it, what works for each person, ability to reflect on past experiences and its effects) [3,34,36,38,39,40], illness/symptom experience [41,42,43], coping and interpersonal skills [33,44], self-care ability [33,43], and self-efficacy [36,37,45].

At the microsystem level, attributes for successful adherence include the quality of the therapeutic alliance, making room for negotiation, and shared decision making [39,40,45,46,47,48,49,50,51,52,53,54,55,56,57]. A patient-centered therapeutic alliance developed on shared-decision making, mediated by the clinician’s experience [49], characterizes therapeutic engagement. Ongoing support of patients and families, family involvement [39,40,48,51,58], and social support from mutual support groups and peer workers [40,46,59,60] reinforce the sense of control with increased awareness of a patient’s right to make choices and engage.

At the meso/exosystem level, addressing therapy-related factors should contribute to improving patient adherence. Preplanned interventions, type of medication regimen, and prescribing mixed intervention approaches might encourage patient adherence [40,50,51,61,62,63,64]. Therefore, to reduce potential therapeutic barriers, HCPs should consider a person-centered approach when developing treatment plans and engaging patients in the process. Factors related to healthcare organizations were also seen to affect adherence. Accessibility, continuity of care, good planning, multidisciplinary approach, and quality of healthcare responses are important attributes of adherence [40,46,48,51,62,65,66].

**Table 1:** Attributes of therapeutic adherence concept.

Individual Level	Microsystem Level	Meso/Exosystem Level
**Motivation for treatment and readiness to change** [34,35,36,37]. **Medication literacy combines:** (a) Readiness to understand the role of medication, how to use it, and understanding how it works for the patient [34,38]; (b) Knowledge about mental health disorders and understanding how to use medication [3,34,38,39]; (c) Understanding what works personally [38]; (d) Ability to reflect on past experiences with medication, self-medication, and forgetfulness [40]. **Illness-Symptom experiences:** (a) Tolerance to side effects of medication [41,42]; (b) Symptoms and severity of symptoms [42,43]. **Personal resources include:** (a) Coping skills [44]; (b) Self-efficacy [36,37,45]; (c) Self-care ability [33,43]; (d) Ability to remember [43]; (e) Ability to establish interpersonal skills [33].	**Quality of the therapeutic alliance** [40,45,46,47,48,49,50,51,52]. **Shared decision making and negotiation** [39,40,46,50,53,54,55,56,57]. **Community support contains:** (a) Family involvement and social support [39,40,48,51,58]; (b) Patient and family ongoing support [42,48,61]; (c) Mutual support groups [46,59,60]; (d) Peer workers as role models [40].	**Therapy-related factors:** (1) Medication plan (a) Type of medication (antipsychotics) [51]; (b) Route of administration/delivery; Alternate oral with injectable medication [40,50,61,62]; (c) Long-acting depot injection reduces nonintentional nonadherence [40,50,63]; (d) Storage of medication-medication packs or dosette boxes [40]/pillboxes [64]. (2) Other Interventions (a) Psychoeducation along with drug treatment [50]; (b) Type of treatment provision (telehealth vs. face-to-face) [52,61]; (c) Use of reminders and electronic prompting [50,62,64]. **Organizational-related factors:** (a) Availability and flexibility of services (schedules) [48,65]; (b) Person-centered planning and collaborative documentation [66]; (c) Community centers and community groups [40]; (d) Services oriented toward their individual goals [66]; (e) Quality of care and attention to patient care needs based on an individual treatment project [65] and case management [40]; (f) Continuity of care [46,51,62] (g) Control of patients over their treatment [66]; (h) Good discharge planning [49]; (i) Home visits [62]; (j) Multidisciplinary work [65].

### 3.3. Antecedents

The iterative process of concept analysis defines antecedents as factors that occur before a patient decides to follow and adhere to the HCP’s recommendations. Therapeutic adherence is defined by two key categories of antecedents: those linked to the patient and those linked to patient–HCP therapeutic interaction.

The first category contains all the patient’s antecedents, such as their background, beliefs and attitudes, and acceptance of mental illness. The background comprises sociodemographic characteristics—namely education [63], age [43,45,62], financial condition [67,68] and employment status [40], and clinical status—such as the course of mental illness [45,50], comorbidity, and mental illness diagnosis [43,69], and substance abuse [50,70]. Beliefs and attitudes refer to individual perceptions regarding spiritual well-being [51], perceived spiritual power [71], religion [51,71], hopes [36,44], and perceived stigma [3,41,60,72]. Acceptance of mental illness encompasses insight about their illness and treatment (level of awareness or understanding) [40,41,42,50,51,58,63,72,73], self-esteem [72,74], self-determination [75], confidence in achieving goals [45], and need for safety and treatment [40,76].

The second category is related to patient-HCP therapeutic engagement and included factors that reported the healthcare provider’s influence [39], how their friendly and non-judgmental behavior facilitates growth [52], how this affects the quality of care of psychiatric patients, and the attitudes of caregivers/relatives/families about the medication and illness [39,73]. This establishes a supportive social network and facilitates therapeutic adherence via a relational-related foundation.

### 3.4. Consequences

Three different consequences emerged from this analysis: an improvement in clinical and social outcomes, commitment to treatment, and the quality of healthcare delivery.

Many articles linked therapeutic adherence and improvement of clinical and social outcomes such as: (a) symptom control [40,49,50,51,62,69,70]; (b) relapsed prevention [33,49,51,63,70]; (c) perceived recovery [36,46,60]; (d) quality of life, well-being, and maintenance of a healthier lifestyle [41,49,72,77]; (e) better functioning level [70]; (f) improved social functioning [63]; and (g) work performance [33,74].

Commitment to treatment is a behavioral strategy explicitly oriented toward mental illness management [36,38,45,48,62,77]. Some articles described therapeutic adherence as a fluid process that reinforces the collaborative approaches between the patient and the clinician [38,56,76] and improves the relationship with professionals [46], affecting satisfaction with treatment [34,45,62,77,78].

Evidence also stresses the pivotal impact of therapeutic adherence on the quality of healthcare delivery, including increased demand for help and adherence to scheduled appointments [49], reduced rates of hospitalizations/rehospitalizations [33,61,63,70], reduction or elimination of sudden and unsupervised treatment interruptions [46], and decreased hospitalization costs [63].

### 3.5. Pragmatic Utility and Empirical Referents

Across the evaluated literature, therapeutic adherence is a challenging concept to assess. Several studies used pill counts [number of dosage units dispensed−number of dosage units remained], laboratory data monitoring and electronic surveillance [79]. Other studies used self-report measures, namely: (a) The Medication Adherence Rating Scale (MARS), a concise instrument for the assessment of medication compliance in psychosis, composed of a 10-dichotomous answer option [37,60,63,64,74,79]. The modified 8-item scale, known as the MARS-8, replaced the dichotomous response (Yes/No) with a 4-point frequency scale (ranging from 1 “Never” to 4 “Always”) to better portray the range of non-adherent behaviors; (b) the Morisky Medication Adherence Scale (MMAS-8), an eight-item structured self-report assessment of medication adherence to help identify barriers and behaviors related to adherence to chronic medications such as psychiatric drugs [51,79]; (c) the Drug Attitude Inventory-10 (DAI-10), which assesses participants’ perception and experience with psychotropic medication [45]; and (d) the treatment adherence checklist, 18 quantitative items measuring treatment adherence and five qualitative items about the reasons for nonadherence as rated by the family caregiver [64].

Psychopharmacological treatment adherence can be considerably improved if strategies suited to each patient’s requirements are used. Self-reported instruments are simple to administer but may add participant bias. All the mentioned tools were used to rate adherence to medication that narrows the multidimensional assessment of therapeutic adherence. New instruments should be created to minimize this problem, particularly instruments involving a multidimensional approach that includes the social and psychological environment.

### 3.6. Exemplar Case Study

After defining the attributes of “therapeutic adherence”, we identified a real-life exemplar case. George is a 22-year-old single man who lives with his parents. He is a university student and works part-time at a gas station to help make ends meet; there is no known personal or family history of mental illness. He has an intense social life, with regular consumption of cannabinoid substances.

In recent months, he began to reveal symptoms such as auditory hallucinations and persecutory delusions, which have been associated with a change in social interaction behavior. Given George’s change in behavior, one of his professors requested support from the University’s health services. He was referred to a Psychiatry consultation and diagnosed with Psychosis. After achieving symptomatic control, he agreed to be referred for multidisciplinary follow-up on an outpatient basis. The treatment program involved an oral home medication regimen, supervised by the parents, and consultations with the Mental Health Nurse (MHN)–case manager–to promote insight through the psychotherapeutic relationship.

With the instituted treatment, the previous symptoms improved. However, side effects of antipsychotic drugs began to appear: drowsiness, lethargy, and decreased libido. These symptoms interfered with social interactions with colleagues and intimate relationships. He felt belittled by colleagues and experienced lower academic and work performance. He decided, therefore, to abandon drug therapy, which led to the resurgence of symptoms, to the point he no longer went to college and work. This situation persisted for more than 6 months.

His concerned parents contacted the nurse case manager, who suggested bringing George for reassessment in consultation. The multidisciplinary follow-up team, with clinical experience, performed a holistic assessment of his situation, focusing on his experience of symptoms, reasons for nonadherence, beliefs and internalized stigma, and validating his experience of mental suffering. The final plan resulted from mediation, consensus and shared decision making between the team and George. At their request, the family was involved in the therapeutic process, initiating individual and family psychoeducation. George was also involved in a mutual help group. Both interventions were managed by the MHN.

A mixed medication plan was negotiated: daily oral and monthly intramuscular. A digital resource with reminders for taking medication was used. When necessary, teleconsultation with the case manager was available. The application of these strategies resulted in continuity and supervision of care, strengthened relationship with professionals, perceived recovery, satisfaction and commitment to treatment, and reduction of costs and time consumption.

Since the introduction of these measures, George has been present in all consultations and the MARS instrument has shown adherence to the therapeutic regimen. The impact of the measures on his functionality and quality of life led him to negotiate with the MHN for greater autonomy in managing his therapeutic regime, suggesting reduced frequency of face-to-face contact, while keeping the option of teleconsultation when necessary.

### 3.7. Operational Definition

**Figure 2:**
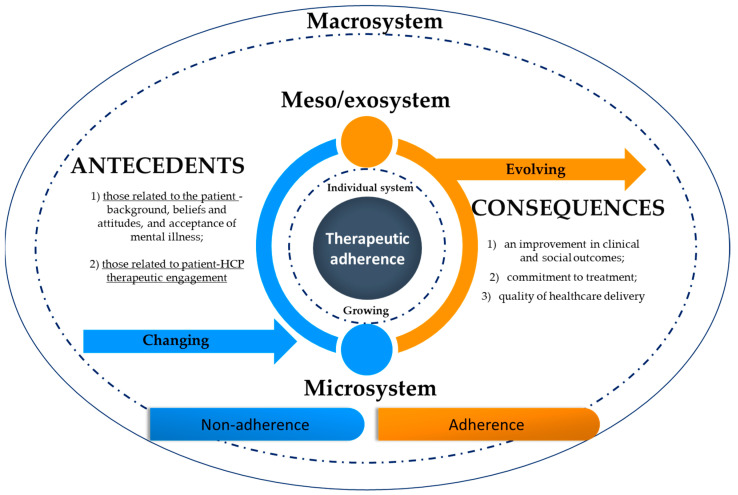
Conceptual representation of therapeutic adherence.

The literature search helped outline the concept and its defining attributes. Consequently, “therapeutic adherence” is defined as the recovery-oriented skills to manage and comply with the therapeutic regimen through self-awareness, knowledge, self-control, and reliance in order to achieve, maintain or promote optimal levels of functioning, health and well-being. These skills are determined by personal, interpersonal, and collective factors that act as facilitators or obstacles. Adherence is constantly in flux; changing, growing, evolving, and transitioning between nonadherence to adherence (see Figure 2).

During this transition between nonadherence and adherence, there may be a boomerang effect, where people choose the opposite of the HCP’s indications because of how they are presented. This effect may result from attempts to change a person’s behavior in a manner that threatens or challenges their worldview. In this sense, improvements in individual motivation and readiness to change, quality of therapeutic alliance, shared decision making, community support, and quality of healthcare delivery may decrease this boomerang effect.

## 4. Discussion

The multifactorial nature of therapeutic adherence has been highlighted in the literature as a facilitator to optimize all the benefits of current therapies. This has implied a shift from the traditional disease-oriented model to a holistic view of health care, centered on the sick individual. Thus emerged the concept of Patient-Centered Care (PCC) [80] that includes shared decision making among all participants in the treatment process, a collaboration between healthcare providers and patients to ensure that decisions fit patients’ preferences, needs and opinions, and that patients have the information and support they need to make decisions and participate in their own care.

Adherence to a prescribed therapeutic regimen is impacted not just by the patient’s individual features but also by variables in the patient’s environment or so-called system-level factors. However, until today, the micro-, meso-, and macro-levels of healthcare system variables have received little attention in explaining therapeutic adherence. Overall, our findings support the notion of person-centered care, highlighting the increased empowerment of people with mental illness, integrated into their biopsychosocial and cultural context [81,82,83]. The core feature of this approach is treating people with compassion, respect and dignity, which empowers them, offers them choices, and involves them in a partnership. This philosophical perspective also emphasizes the importance of an integrative approach where the role of the professional, as a case manager, is central to the optimization of these assumptions [84]. In this sense, recovery is closely linked to the person’s empowerment, described as a deeply personal process of change in attitudes, values, feelings, goals, abilities, and/or functions. It is a way of living a life of satisfaction, hope, and contribution [85]. This process involves developing new meaning and purpose in life based on the knowledge and acceptance of mental illness. The promotion of adherence to the therapeutic regimen based on the philosophy of recovery presupposes that the HCP put aside the paternalistic biomedical model and adopt a posture centered on patient needs and values, allowing patients to play an active role in the adaptive process inherent to the disease trajectory [86]. Nevertheless, therapeutic relationships are commonly based on paternalistic views by both HCPs and users, especially when insight or acceptance of mental illness is absent [76].

As PCC is the philosophy of inherence, specific individual characteristics should be considered for the quality of the therapeutic relationship, and this might be challenging for HCP. Improving competencies in motivational techniques, empowerment, and hope management might be crucial to help patients grow and change [87,88]. This implies that patients advance from one initial stage (changing) to another (growing) in their evolution and commitment to a therapeutic regimen. This also supports the hypothesis that people living with a chronic mental illness who require medication and psychotherapeutic interventions on a regular basis might gain experience in daily self-management (evolving).

In addition to patient attributes, such as motivation and readiness to change, medication literacy, illness–symptom experiences, and availability of personal resources, other relational and organizational attributes of adherence were found. Connection and alliance with patients allow them to better understand their symptoms and achieve therapeutic adherence, which in turn helps stabilize their symptoms and reduce the burden on family members [89]. At the same time, family carers should also be integrated into this process.

In addition to the significance of specific care plans, patients place a high value on therapeutic connections with peers or mental health professionals in community settings, believing that these are essential to recovery. These complex processes can be hindered by the nature of organizational structures and positions that limit the care HCPs can offer [90]. Evidence indicates a need to invest in the creation of multimodal, flexible therapeutic plans focused on user needs. Additionally, organizations must promote good practices that are simultaneously multidisciplinary and structured in a time sequence capable of guaranteeing the gradual acquisition of skills. Only then will it be possible to contribute to the improvement of clinical and social outcomes, generate commitment, and guarantee a quality of services that translates into increased adherence rates and reduced number of hospitalizations and all associated costs (including pharmacy costs and inpatient and outpatient costs) [91]. Furthermore, a person-centered approach predicts better outcomes, such as fewer relapses, suicide and overall mortality [91].

In contrast, the impact of subjective norms on adherence and perceived social pressures (macrosystem realm) was not understood in this research. This might be attributed to variations in cultural norms and beliefs. Western cultures, for example, are often individualistic, and people from these cultures may be more driven to behave in accordance with their own goals and views. Meanwhile, in non-Western societies, social pressures are determinants of therapeutic adherence behaviors [21]. This might be due to the collectivistic ethos of these communities, which have always encouraged interconnection with others. Thus, social effects often explain the public stigma surrounding mental health illnesses, which hampers people’s intent to seek assistance and adhere to therapy regimens for fear of social rejection [21]. Therapeutic adherence is expected to improve in patients with mental illnesses who typically require supervision and close help, as community-based services are increasingly strengthened. Governments should create local community-based mental health services, given the relevance of psychiatric community rehabilitation in treatment adherence [3].

Finally, during the dynamic process of adherence, we should recognize the boomerang effect opposing the intended intervention. Several factors could contribute to this negative effect, namely: (a) when the message content is too far removed from the patient’s position, (b) when no good arguments are contained in a delivered message, (c) when the patients anticipate that the conclusions will be contrary to their interests, and (d) when communication induces emotional arousal and aggressive behavior [92]. The potential costs of these boomerang effects, their causes, and their individual variations have received little consideration in the extant research. Nonetheless, HCPs should be aware of this issue, and cultivate relationship skills and embrace the risk of unintentional boomerang consequences in their clinical practice.

### 4.1. Study Limitations

This concept analysis has several limitations. First, although the search was systematic, only two databases were used, and relevant papers may have been missed. However, its systematic structure facilitates the use of results by stakeholders interested in applying the findings to healthcare policy and practice. Second, the exploration of the use and influence of therapeutic adherence across related healthcare disciplines was not examined, as the studied articles mainly focused on nursing. Thus, Rodgers’ [15] suggestion to include 30 papers or 20% from each area in the study could not be implemented. A further limitation is present in the current literature that clearly highlights the first three interconnected levels of therapeutic adherence behavior (patient, microsystem and meso/exosystem levels). What is less clear is the influence of societal and cultural ideologies and values on that behavior, because analyzed studies do not offer information about the macrosystem sphere. Furthermore, there is a paucity of standardized instruments that measure therapeutic adherence in the mental health context. This restricts our confidence in the accurate measurement based on an ecological perspective. Finally, only adult patients and papers published in English or Portuguese were studied.

### 4.2. Implications

The formulation of implications that can drive further concept development is a key outcome of Rodgers’ method. Our concept analysis reveals persistent barriers to participants’ process of adhering. Although adherence to therapy is sensitive to different disciplinary areas, mental health nursing interventions that address the dynamic process of adherence can be designed to assist patients in achieving a seamless transition throughout stages (from nonadherence to adherence). Patients are frequently in emotional discomfort and overwhelmed by the information and constraints provided during the early period. As a result, nursing should focus on improving psychoeducation of mental diseases to address individual patients’ concerns, discuss misconceptions, and relieve anxiety to support coping and change. Several tactics might be employed, namely: building a trusting connection with professionals, involving family caregivers, assessing nonadherence, providing self-management information, addressing medication concerns, and fostering comprehension and empowerment [39,88]. Afterward, patients tend to practice their own level of adherence to living with mental health conditions, exhibiting personal improvement.

According to the WHO’s [5] approach to promoting patients’ ability to self-manage chronic diseases, MHNs should collaborate with patients to identify areas where flexibilities and changes are available and enable patients to live life as normally as possible. One strategy to enhance engagement is to provide culturally sensitive and competent care. While the macrosystem is still unclear, we assume that a person’s culture and contextual history will influence how they perceive mental illness, therapy, and engagement with the treatment team. These concerns should be researched further.

In the long term, evolving and positive reinforcement of adherence should be maintained. Patients who are successful in self-management might be invited to share with other patients their own experiences dealing with life changes. This can help patients who are new to the dynamic process of adherence and boost the self-esteem of those who have been through it. As a result, nurses should modify care delivery depending on the continuing patient evaluation. Since MHNs have the most direct contact with patients, they play a critical role in ensuring that patients adhere to long-term therapy. Improving adherence can improve therapeutic efficacy, clinical practice development, medical expenses, and patient safety.

## 5. Conclusions

When all the attributes are present in therapeutic adherence behavior—individual, microsystem, and meso/exosystem levels—the full meaning of the concept is translated into a comprehensive care practice. This framework emphasizes the complexities of therapeutic adherence in the context of mental health and illustrates its dynamic capacity to span many disciplines. Within the current literature, the findings imply a comprehensive definition of therapeutic adherence addressing a holistic picture of the patient and their circumstances.

## Data Availability

All data analyzed during this study are included in this article.
